# Fish population genetic structure shaped by hydroelectric power plants in the upper Rhine catchment

**DOI:** 10.1111/eva.12339

**Published:** 2016-01-08

**Authors:** Alexandre Gouskov, Marta Reyes, Lisa Wirthner‐Bitterlin, Christoph Vorburger

**Affiliations:** ^1^ Institute of Integrative BiologyETH ZürichZürichSwitzerland; ^2^ Aquatic EcologyEawagDübendorfSwitzerland; ^3^ Life Sciences and Facility ManagementZHAWWädenswilSwitzerland

**Keywords:** conservation, fragmentation, population genetics, *Squalius cephalus*

## Abstract

The Rhine catchment in Switzerland has been transformed by a chain of hydroelectric power stations. We addressed the impact of fragmentation on the genetic structure of fish populations by focusing on the European chub (*Squalius cephalus*). This fish species is not stocked and copes well with altered habitats, enabling an assessment of the effects of fragmentation *per se*. Using microsatellites, we genotyped 2133 chub from 47 sites within the catchment fragmented by 37 hydroelectric power stations, two weirs and the Rhine Falls. The shallow genetic population structure reflected drainage topology and was affected significantly by barriers to migration. The effect of power stations equipped with fishpasses on genetic differentiation was detectable, albeit weaker than that of man‐made barriers without fishpasses. The Rhine Falls as the only long‐standing natural obstacle (formed 14 000 to 17 000 years ago) also had a strong effect. Man‐made barriers also exacerbated the upstream decrease in allelic diversity in the catchment, particularly when lacking fishpasses. Thus, existing fishpasses do have the desired effect of mitigating fragmentation, but barriers still reduce population connectivity in a fish that traverses fishpasses better than many other species. Less mobile species are likely to be affected more severely.

## Introduction

The ongoing landscape modification by humans leads to massive destruction or alterations of pristine ecosystems by a combination of fragmentation, habitat loss and degradation (Sala et al. 2000; Foley et al. 2005; Fischer and Lindenmayer 2007). In riverine ecosystems, fragmentation is considered a key threat for aquatic biodiversity because organisms are restricted to linear dendritic habitats and cannot avoid anthropogenic barriers (Fagan 2002; Vörösmarty et al. 2010). Despite the recognized importance of fragmentation in aquatic conservation, the fragmentation literature is currently biased towards terrestrial ecosystems (Fazey et al. 2005).

River catchments suffered from heavy floodplain losses of up to 90% in the USA and even more than 90% in Europe, as for example in Switzerland where 95% of the floodplains have been lost (Tockner and Stanford 2002). Concurrent with this destruction, the same regions are also the most fragmented by dam‐building (Tockner and Stanford 2002; Nilsson et al. 2005; Lehner et al. 2011). An inevitable consequence of the many barriers in rivers has been that currently, diadromous fish species are the most threatened at the global (Liermann et al. 2012) and local scale (Kirchhofer et al. 2007). Rieman and Dunham (2000) reviewed the situation for salmonids that are structured in metapopulations and concluded that river fragmentation is frequently the reason for population collapse.

Since the early days of dam construction, fishpasses have been constructed to mitigate negative effects on fish migration (Katopodis and Williams 2012). Noonan et al. (2012) published a meta‐analysis of fishpass efficiency assessments from 1960 to 2010. They found 122 articles published over 50 years reporting such assessments – astonishingly few considering the high costs of fishpasses – and most of these assessments focused on salmonids. The conclusions were that the design of fishpasses affected their efficiency and that they worked better for salmonids than for other fish, but that the overall efficiency was too low to avoid the negative effects of habitat fragmentation on the fish community (Noonan et al. 2012). The focus on salmonids is warranted due to their economic value and their life histories making them particularly vulnerable to fragmentation (e.g. anadromous salmon, trout and char forms). Nevertheless, even nonmigratory species require between 1 to 100 km river length for their entire life history and at this scale habitat changes due to major flooding events occur in the order of every 5–50 years (Fausch et al. 2002). It is thus important that fishpasses are designed to also facilitate the migration of fish other than salmonids and that their efficiency in doing so is evaluated.

The long‐term persistence of species depends on sufficient genetic diversity to adapt and survive in variable or changing environments (Hughes et al. 2008). If local populations are small, gene flow is the key factor to prevent the stochastic loss of genetic diversity (Palstra and Ruzzante 2008) and to provide the required alleles to subpopulations under selection that lack favourable genotypes (Kinnison and Hairston 2007). While an effective population size of just 50 may be sufficient to avoid the negative effects of inbreeding in the short term, the long‐term maintenance of adaptive potential requires an effective population size in the range of at least 500 (Franklin and Frankham 1998; Jamieson and Allendorf 2012). Some authors argued that the required number may be even higher (Lynch and Lande 1998). Unfortunately, the ratio between the effective and the total population size is difficult to predict and often species specific in freshwater fish. Published values range from <0.01 for introduced bottlenecked pike (*Esox lucius* Linnaeus) (Aguilar et al. 2005) over intermediate values like 0.11 in natural population of brown trout (*Salmo trutta* Linnaeus) (Charlier et al. 2011) to very high ratios approaching 1 in the case of the endangered copper redhorse (*Moxostoma hubbsi* Legendre) (Lippe et al. 2006). Therefore, fishpass efficiency is not simply a matter of the number of climbing or descending fish. The goal has to be to allow sufficient gene flow for a species to maintain its evolutionary potential in a fragmented habitat.

Previous studies investigated fragmentation caused by barriers that were impassable for fish in the upstream direction, and these studies showed that such barriers had a strong impact on the genetic population structure. Examples include yellow perch (*Perca flavescens* Mitchill) (Leclerc et al. 2008), Macquarie perch (Faulks et al. 2011), brown trout (Horreo et al. 2011; Stelkens et al. 2012), bullhead (*Cottus gobio* Linnaeus) (Hanfling and Weetman 2006; Junker et al. 2012), three‐spined stickleback (*Gasterosteus aculeatus* Linnaeus) (Raeymaekers et al. 2008), grayling (*Thymallus thymallus* Linnaeus) (Meldgaard et al. 2003), chub (*Squalius cephalus* Linnaeus) (Dehais et al. 2010) and a four species comparison of chub, dace (*Leuciscus leuciscus*), gudgeon (*Gobio gobio*) and minnow (*Phoxinus phoxinus*) by Blanchet et al. (2010).

Here we present a large‐scale study on the effects of dams in a strongly fragmented system where a large proportion (89%) of dams enable migration through fishpasses. We selected the common chub *(Squalius cephalus* Linnaeus) as our model species, a European cyprinid species that is very common in the Swiss midlands, where it lives in high number in rivers of the Barbel and Grayling Region [categorization according to Huet (1949)] as well as in lakes (Zaugg et al. 2003). Chub reach an average length of 40–50 cm in the study region (Zaugg et al. 2003). Due to their ecological generalism, omnivory and behavioural flexibility, chub cope relatively well with the ongoing habitat alteration. For spawning grounds, they prefer shallow gravel banks (0.1–1.0 m) with some current (0.15–0.35 m/s) (Fredrich et al. 2003). They readily use alternative spawning grounds in altered habitats, as long as some stony bottom is available (Arlinghaus and Wolter 2003). Chub are relatively mobile and show an upstream spawning migration of up to 16 km (river Spree, Fredrich et al. 2003) or even in excess of 25 km (River Meuse, De Leeuw and Winter 2008). Chub are iteroparous, spawn twice a year and may migrate to different spawning grounds between spawnings (Fredrich et al. 2003). The upstream spawning migration may compensate for downstream drift in the larval stage. Chub larvae actively swim into open water to go into drift, which is a common behaviour in many cyprinid species (e.g. Reichard et al. 2002; Reichard and Jurajda 2004; Sonny et al. 2006). Female chub reach maturity in their second to fourth year (Raikova‐Petrova et al. 2012) and can reach a maximum age of up to 20 years (Busst and Britton 2014). Because of their low commercial value, chub are not stocked. The genetic structure of chub populations should thus be unconfounded by stocking, making this species especially useful as a sentinel for changes in population connectivity resulting from obstructions to dispersal.

In this study, we describe the genetic population structure of the chub in the main Swiss lowland rivers of the Rhine catchment and assess the effects of barriers such as hydroelectric power plants on population connectivity and genetic diversity. In particular, we ask whether fishpasses that are present at most (but not all) barriers do have the desired effect of mitigating fragmentation.

## Methods

### Study area

**Figure 1:**
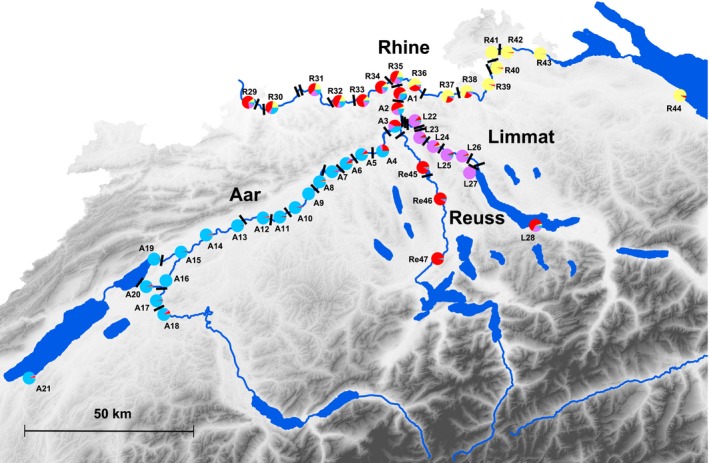
Map of the Swiss midland with the rivers Rhine, Aar, Reuss and Limmat. Pie charts depict mean assignment probabilities of chub genotypes to each of the four inferred genetic clusters, averaged over 28 TESS runs. Black bars represent migration barriers. These are hydroelectric power stations except for the Rhine falls (between site R40 and R41), and two weirs (represented as one bar between L26 and L27).

Our study area comprises the larger Swiss midland rivers of the upper Rhine catchment (length 376 km, catchment area 35 897 km^2^, discharge 1037 m^3^/s). In addition to the Rhine, the Rhine catchment includes the river Aar (length 288 km, catchment area 17 620 km^2^, discharge 560 m^3^/s) with its two tributaries Reuss (length 158 km, catchment area 3425 km^2^, discharge 140 m^3^/s) and Limmat (length 140 km, catchment area 2416 km^2^, discharge 101 m^3^/s) (Verdon et al. 2009) (Fig. 1). The first dam was built in 1830 for textile manufacturing, followed by many hydroelectric power stations from 1861 to 1975. At present, the studied river sections are fragmented by 37 hydroelectric power stations, of which four do not have a fishpass, and two adjacent weirs also lacking a fishpass. The two adjacent weirs are considered as a single barrier in all analyses. When fish sampling started in 2010, the median age of the artificial obstacles was 82 years and that of the fishpasses 77 years (age known for 23 fishpasses, for all others information was not publicly available) (Table S1 and Fig. S1). The Rhine Falls are the only natural barrier in the study area and estimated to be between 17 000 and 14 000 years old (Fig. 1). According to monitoring data from the hydroelectric power stations, chub are able to use all fishpasses in the study area (Guthruf 2006, 2008).

### Site selection and fish sampling

Chub from 47 sites were sampled from spring to autumn 2010 and 2011 in the Rhine, Aar, Reuss and Limmat catchments, which include also three lakes (Fig. 1). The goal was to sample each fragment, defined as a river section between two barriers, at least once, and to sample two or more sites from some fragments to break the pairwise correlation between waterway distance and the number of barriers between sampling sites. The fish were caught by electro fishing (FEG 1700; EFKO comm., Leutkirch, Germany), wading along the river shoreline. The exact locations were chosen in consideration of accessibility and safety. At three sites, we could not catch in the main river but managed to obtain samples from the mouths of tributaries (A13, L27, R29). At two sites where electrofishing was impossible, fish were caught with rod and line (sites A19 and L28). Sampling site coordinates are provided in Table 1. The goal was to catch 50 chub per site which was achieved at most sites. Most individuals caught were young of the year (70%), the remaining fish were juveniles and a few adults. Because chub undergo a larval drift phase (see Introduction), we do not expect the fish from a single sampling site to be related (e.g. siblings). Clove oil was used to lightly anaesthetize the fish before total length measurement and tissue sampling from the caudal fin. Fin clips were stored in 99.9% ethanol, and all fish were released at the sampling sites after recovering from anaesthesia.

**Table 1:** Collection information and measures of genetic diversity for 47 samples of chub (*Squalius cephalus*) from the Swiss Midland rivers Aar (A), Limmat (L), Rhine (R), and Reuss (Re). *N*: sample size, *H*_*e*_: expected heterozygosity; *H*_*o*_: observed heterozygosity; AR*:* allelic richness standardized for the smallest sample size (20), *F*_IS_: inbreeding coefficient; *N*_*e*_: effective population size

Site	Coordinates WGS84	*N*	*He*	*Ho*	*F*_IS_	AR	*Ne* estimate (95% CI)
A1	N 47°33′32.97″E 8°13′53.06″	50	0.748	0.753	−0.007	7.126	∞ (301–∞)
A2	N 47°32′38.45″ E 8°13′46.80″	50	0.737	0.724	0.017	6.934	439 (118–∞)
A3	N 47°29′07.19″ E 8°12′58.84″	48	0.747	0.742	0.007	7.244	∞ (641–∞)
A4	N 47°25′03.88″ E 8°09′43.08″	48	0.728	0.709	0.026	7.034	351 (69–∞)
A5	N 47°24′29.97″ E 8°04′01.72″	49	0.744	0.755	−0.014	7.632	205 (91–∞)
A6	N 47°23′01.72″ E 8°00′50.45″	50	0.746	0.719	0.035	7.155	∞ (349–∞)
A7	N 47°21′57.55″ E 7°59′37.84″	49	0.739	0.745	−0.007	7.172	629 (174–∞)
A8	N 47°18′53.87″ E 7°53′29.30″	49	0.734	0.750	−0.022	7.115	283 (113–∞)
A9	N 47°18′37.57″ E 7°52′09.98″	25	0.756	0.769	−0.017	7.274	∞ (121–∞)
A10	N 47°15′49.35″ E 7°48′24.77″	50	0.728	0.747	−0.026	6.637	401 (110–∞)
A11	N 47°14′16.29″ E 7°44′42.19″	49	0.734	0.750	−0.021	7.249	3962 (111–∞)
A12	N 47°14′07.67″ E 7°40′37.44″	54	0.746	0.761	−0.021	7.216	369 (132–∞)
A13	N 47°12′51.54″ E 7°34′24.28″	28	0.753	0.732	0.028	6.869	∞ (602–∞)
A14	N 47°11′19.92″ E 7°26′51.32″	48	0.723	0.715	0.012	6.736	551 (113–∞)
A15	N 47°08′30.69″ E 7°20′46.29″	36	0.727	0.734	−0.009	6.977	∞ (1893–∞)
A16	N 47°02′44.27″ E 7°16′28.32″	50	0.729	0.748	−0.027	6.534	∞ (264–∞)
A17	N 47°00′24.12″ E 7°14′49.34″	54	0.727	0.740	−0.018	6.822	202 (93–∞)
A18	N 46°58′24.58″ E 7°15′35.76″	36	0.714	0.739	−0.035	6.812	651 (104–∞)
A19	N 47°07′20.34″ E 7°14′13.74″	32	0.720	0.677	0.060*	6.669	232 (74–∞)
A20	N 47°02′47.60″ E 7°12′21.73″	42	0.725	0.711	0.020	6.715	8270 (96–∞)
A21	N 46°47′35.08″ E 6°44′17.48″	49	0.639	0.665	−0.041	5.851	∞ (160–∞)
L22	N 47°29′03.39″ E 8°17′25.28″	50	0.713	0.721	−0.011	6.192	59 (40–99)
L23	N 47°27′22.38″ E 8°18′45.74″	50	0.724	0.720	0.005	6.176	125 (72–345)
L24	N 47°24′31.51″ E 8°24′37.39″	48	0.715	0.666	0.069*	6.107	50 (25–164)
L25	N 47°24′16.86″ E 8°26′09.85″	50	0.701	0.698	0.004	5.812	187 (79–∞)
L26	N 47°24′03.46″ E 8°29′05.16″	49	0.711	0.676	0.049*	5.851	∞ (101–∞)
L27	N 47°20′54.09″ E 8°30′57.75″	48	0.660	0.654	0.010	4.720	69 (34–281)
L28	N 47°12′26.98″ E 8°46′35.03″	30	0.695	0.688	0.010	6.504	∞ (127–∞)
R29	N 47°33′12.70″ E 7°37′6.017″	48	0.727	0.730	−0.004	6.761	169 (69–∞)
R30	N 47°32′19.35″ E 7°42′51.34″	47	0.742	0.747	−0.006	7.787	∞ (280–∞)
R31	N 47°35′15.08″ E 7°53′16.80″	50	0.738	0.769	−0.042	7.414	474 (183–∞)
R32	N 47°33′17.43″ E 7°59′14.33″	49	0.736	0.701	0.048*	8.178	256 (136–1372)
R33	N 47°33′24.52″ E 8°05′01.38″	48	0.730	0.710	0.027	7.358	1694 (114–∞)
R34	N 47°35′34.95″ E 8°09′38.66″	50	0.728	0.702	0.036	7.241	165 (79–3223)
R35	N 47°36′23.24″ E 8°13′21.66″	56	0.736	0.732	0.006	7.690	1114 (214–∞)
R36	N 47°35′55.13″ E 8°17′43.24″	49	0.706	0.692	0.020	7.381	264 (88–∞)
R37	N 47°33′59.08″ E 8°25′43.14″	49	0.712	0.712	0.001	7.269	763 (182–∞)
R38	N 47°34′42.14″ E 8°30′20.53″	53	0.725	0.715	0.014	7.226	548 (125–∞)
R39	N 47°35′49.36″ E 8°35′44.49″	49	0.669	0.655	0.021	7.502	∞ (322–∞)
R40	N 47°39′09.40″ E 8°37′46.12″	33	0.658	0.653	0.008	7.027	314 (62–∞)
R41	N 47°41′05.50″ E 8°37′35.20″	50	0.651	0.625	0.040	6.763	1274 (128–∞)
R42	N 47°41′05.17″ E 8°40′22.69″	48	0.661	0.651	0.016	7.578	250 (90–∞)
R43	N 47°40′43.48″ E 8°48′25.57″	26	0.667	0.671	−0.006	7.057	130 (52–∞)
R44	N 47°33′22.20″ E 9°21′58.62″	21	0.721	0.734	−0.018	7.908	∞ (2538–∞)
Re45	N 47°22′16.54″ E 8°19′29.01″	49	0.735	0.725	0.014	6.998	1216 (161–∞)
Re46	N 47°17′02.82″ E 8°23′35.59″	39	0.742	0.751	−0.012	6.651	1694 (177–∞)
Re47	N 47°07′07.11″ E 8°23′26.11″	48	0.730	0.725	0.007	6.602	437 (83–∞)

*P < 0.05.

### Genotyping

DNA was extracted following the salting‐out protocol of Sunnucks and Hales (1996) adapted to a 96 deep well format. Fin clips (1 mm^2^) were air dried in 8‐strip microtubes before adding 300 *μ*L of TNES buffer **(**50 mm Tris, pH 7.5, 400 mm NaCl, 20 nm EDTA, 0.5% SDS) and 5 *μ*L of 10 mg/mL proteinase K (Roche inc., Basel, Switzerland). After incubation for 60 min in an incubation shaker (Thermomixer Comfort, Eppendorf inc, Hamburg, Germany) at 300 rpm, the proteins were precipitated by adding 85 *μ*L of 5 m NaCl and shaking the tubes for 10 s. Proteins were pelleted in a centrifuge (Heraeus Megafuge 40R; Thermo Fisher Scientific inc, Waltham, MA, USA) at 4700 rpm for 10 min. The supernatants were carefully transferred into a 96‐deep‐well block. Afterwards, the DNA was precipitated by adding 400 *μ*L of ice‐cold 100% ethanol and gentle mixing with a pipette. The DNA was pelleted by centrifugation for 10 min at 4700 rpm, washed with 70% ethanol, centrifuged again for 10 min at 4700 rpm and air dried. Finally, the DNA was resuspended in 100 *μ*L of TE buffer (100 mm Tris‐HCl, 10 mm EDTA) and stored at −20°C.

Ten microsatellite loci amplified in two multiplex PCR were used to genotype the individuals: LC128, LC27, LC290, LC32, LC93 (Vyskocilova et al. 2007), LceA149, LceC1, LceCb (Larno et al. 2005) and N7G5, N7K4 (Mesquita et al. 2003). The 10 *μ*L PCR contained 5 *μ*L Qiagen Multiplex PCR Master Mix (Qiagen inc., Hilden, Germany), 3 *μ*L of ultrapure water, 1 *μ*L of DNA template and 1 *μ*L of primer mix. The forward primers for the two multiplex reactions were labelled with fluorescent dyes (Microsynth inc., Balgach, Switzerland), such that loci with the same dye had nonoverlapping allele size ranges. To balance peak heights, labelled forward primers of strongly amplifying loci were partially supplemented with unlabelled primer. Multiplex 1 contained the following primers (only forward primer concentrations given, reverse primer concentrations were equivalent to the sum of labelled and unlabelled forward primers): N7K4 0.4 nm labelled (YYE) and 14.5 nm unlabelled, N7G5 0.9 nm labelled (AT550) and 30.5 nm unlabelled, LC128 4.7 nm (AT550), LC 27 1.8 nm (FAM), LC 290 1.2 nm (YYE). Multiplex 2: LceA149 0.5 nm labelled (FAM) and 10 nm unlabelled, LC32 0.8 nm labelled (AT565) and 13.7 nm unlabelled, LceC1 1.6 nm (AT550), LceCb 6.8 nm (FAM), LC93 1.9 nm (AT565).

PCR thermal conditions were as follows: initial *Taq* polymerase activation and denaturation at 95°C for 15 min, followed by 30 cycles of denaturation at 94°C for 30 s, annealing at 58°C (multiplex 1) or 57°C (multiplex 2) for 90 s, extension at 72°C for 90 s, followed by a final extension at 72°C for 10 min (Labcycler Sensoquest, Göttingen, Germany).

PCR products were analyzed on an ABI 3730 capillary sequencer (Applied Biosystems, Foster City, CA, USA). For loading, 1 *μ*L of PCR product was mixed with 8 *μ*L Hi‐Di Formamide and 1 *μ*L size standard diluted 1: 7 (GeneScan 500 LIZ, ABI).

The genotyping accuracy was tested by extracting and genotyping a subset of samples twice. Locus LC290 showed a dropout of large alleles, due to its wide allele size range (allele size 223–309 bp). To avoid genotyping errors resulting from this problem, all homozygotes with low peak heights were reamplified with more cycles and scored again. All other loci had scoring errors below 1%. Nevertheless, the scoring was performed three times to minimize mistakes. For the final data set, only individuals that were genotyped successfully at nine of the ten loci at least were considered (132 of 2133 genotyped individuals had one missing locus). That resulted in an average of 45 individuals per site. For the sample sizes per site, see Table 1.

### Population genetic analyses

Estimators of genetic diversity such as observed heterozygosity (*H*_*O*_), expected heterozygosity (*H*_*E*_), mean number of alleles and allelic richness (AR) standardized for smallest *N* were calculated using FSTAT 2.9.4 (Goudet 2002) (Table 1). We tested for the presence of null alleles with the software Micro‐Checker (Van Oosterhout et al. 2004). FSTAT was also used to test for deviations from linkage and Hardy–Weinberg equilibrium (for *F*_IS*,*_ see Table 1). Pairwise population differentiation (*F*_ST_) was calculated according to Weir and Cockerham (1984) as implemented in FSTAT 2.9.4, using 10 000 bootstraps for significance tests. The use of *F*_ST_ to infer population structure has been criticized because of its dependency on within‐population diversity, and alternative measures such as *F’*_ST_ or *D* have been proposed (Hedrick 2005; Jost 2008). However, we decided to focus on *F*_ST_ because it is the appropriate measure of deviations from panmixia (Whitlock 2011), which is our main interest in the context of potential barriers to fish migration. The software NEESTIMATOR v. 2.0.1 (Do et al. 2014) was used to obtain point estimates of the effective population size *N*_e._ We used the method based on linkage disequilibrium restricted to alleles with frequencies >0.02 as recommended by Do et al. (2014). Confidence intervals were obtained with the jackknife method of Waples and Do (2008). To obtain a general overview of the genetic structuring of chub in the Swiss midland rivers, we used the individual‐based Bayesian clustering approach implemented in TESS 2.3.1 (Chen et al. 2007). Neighbouring individuals are more likely to belong to the same genetic cluster, and TESS uses the Hidden Markov Random Field approach to take into account the spatial distribution of individuals (Francois et al. 2006). The interaction parameter *ψ* was set at the default value of *ψ *= 0.6 for the analyses. *ψ* can range from zero (no spatial interaction) to one (strongest spatial interaction). The neighbouring system is carried out by Voroni tessellation (Guillot et al. 2009), which requires individual geographic coordinates for each genotype. To obtain these, the coordinate creator implemented in the software was used. It randomly samples from a normal distribution around the initial coordinate of the population sample. A standard deviation of 25 m was used. As fish can only have up‐ or downstream neighbours, the automatically generated neighbour system was changed to reflect the dendritic network of our river system. This was carried out with the neighbourhood modifying option. All overland links were deleted, and missing links were added (Neighbourhood TESS 2.3.1 output see Fig S2.).

Using the no‐admixture model, each number of genetic clusters (*K*_max_) was tested with 20 runs, for *K*_max_ **= 2–9. The total number of sweeps per run was 300 000 after a burn in of 50 000 sweeps. The best‐supported number of genetic clusters was determined by plotting the mean Deviance Information Criterion (*DIC*) of the 20 runs over the respective *K*_max_ (Spiegelhalter et al. 2002). The best‐supported *K*_max_ is at the point where the *DIC* curve switches from a sharp decrease to a plateau without further changes (for details, see the TESS2.3.1 Manual). Best *K*_max_ was run an additional 20 times. For averaging the results and taking into account ‘label switching’, the programme CLUMPP 1.1.2 (Jakobsson and Rosenberg 2007) was used. The calculations were performed using the Greedy algorithm in CLUMPP 1.1.2. The cluster visualization was performed with the software DiSTRUCT (Rosenberg 2004).

Attempts to estimate contemporary gene flow among sampling sites using the software MIGRATE‐N (Beerli and Felsenstein 2001) failed due to nonconvergence, presumably because of the very shallow overall population structure of the chub in the study area (see Results), and for the same reason inferring recent migration rates with BAYESASS (Wilson and Rannala 2003) produced nonsensical results. It appears that a global *F*_ST_ of >0.05 would be required to use this approach profitably (Meirmans 2014). This requirement of at least moderately strong population structure combined with the requirement of individuals with migrant ancestry being frequent enough to be included in realistic sample sizes may well be a general limitation of this approach and could possibly explain the alarming observation by Meirmans (2014) that most of the published estimates of the proportion of nonmigrants produced by BAYESASS cluster at the upper and lower bounds of the prior distribution.

### Fragmentation effects

Our main interest was to investigate the impact of barriers on population structure and genetic diversity. First, we assessed the general impact of barriers, treating all barriers equally, and second, we tested whether different types of barriers had different effects, focusing particularly on the presence or the absence of fish ladders.

The overall impact of barriers on genetic differentiation was assessed using an isolation‐by‐distance (IBD) approach, correlating the matrix of pairwise *F*_ST_ (*n *=* *1081 pairwise comparisons) with the matrix of pairwise waterway distances extracted with ArcMap10 (Esri inc., Redlands, CA, USA) between sampling sites and the matrix of the pairwise number of barriers (barrier count) between sites. Partial Mantel tests were carried out according to Smouse et al. (1986), using the software Arlequin 3.5 (Excoffier and Lischer 2010) with 10 000 permutations. The Partial Mantel test assesses the effect of the number of barriers between sites on pairwise differentiation while controlling for pairwise waterway distance.

Assessing the impact of different barrier types on gene flow is important for assessing the effectiveness of the fish ladders. To evaluate the impact of the different types of barriers, we split the 39 barriers in our system into different groups: the Rhine Falls as the only major natural barrier (14 000–17 000 years old), hydroelectric power stations with fishpass (*n *=* *33), and power stations without fishpass (*n* = 5). The last group comprised one barrier (the two adjacent weirs) that was not a power station but was included because it could be assumed to be equally obstructive to fish migration. The age of artificial barriers could also influence the differentiation among fish populations they separate. However, we found that the pairwise number of artificial barriers separating two sampling sites and their cumulative age were strongly correlated (*r *=* *0.97). This collinearity precluded an independent assessment of the age of barriers, thus we restricted our analyses on the number of barriers of each type. We constructed six linear models for comparison that had pairwise *F*_ST_ as the dependent variable and the following predictors:

1. distance
2. distance + barrier count
3. distance + count of barriers without fishpass (including Rhine Falls) + count of barriers with fishpass
4. distance + Rhine Falls
5. distance + Rhine Falls + count of all other barriers
6. distance + Rhine Falls + count of barriers with fishpass + count of barriers without fishpass

The relative support for the different models was assessed by model selection using the AIC criterion (Burnham and Anderson 2002), and we followed Koizumi et al. (2006) in using the number of populations (47) rather than the number of pairwise comparisons (1082) as *n* in the calculation of AIC to account for nonindependence inherent in pairwise data. R 2.15.1 was used to perform the analyses (R Core Team 2012). The significance of individual parameters in the models was evaluated with permutation tests using the R package lmPerm (Wheeler 2010). Because of the unavoidable collinearity between distance and the counts of the different barrier types between sampling sites, we applied commonality analysis to the best‐supported models (Nimon et al. 2008), using the R package MBESS (Kelley and Lai 2012). This approach was recently advocated for landscape genetics by Prunier et al. (2015) and allows an assessment of the extent to which individual predictors contribute via unique and shared effects to the explained variance in the response variable.

To complement these analyses and to account for the dendritic structure of the river network, we also applied the STREAMTREE algorithm by Kalinowski et al. (2008) to map genetic differences among chub subpopulations onto the stream sections connecting them. This approach uses least‐squares estimation to model pairwise genetic distance (here *F*_ST_ calculated by the STREAMTREE software) as the sum of genetic distances for the stream sections between them. The coefficient of determination *R* ^2^ is used to assess the fit of the resulting model to the data. Because a canal between the Aare river and Lake Biel (see site A20 in Fig. 1) creates a loop in the drainage structure that is incompatible with the STREAMTREE model, we had to exclude sites A16‐A21 from this analysis.

The genetic diversity expressed as allelic richness (AR) was investigated with a similar model selection approach as used for genetic differentiation. Six linear models of AR were constructed with the same predictors as above, but here distance was calculated from an arbitrary reference point below the most downstream site, namely where the Rhine crosses the Swiss border, and the numbers of the different types of barriers were counted along the river(s) between this reference point and each site.

## Results

### Microsatellite variation

The initial analysis with Micro‐Checker indicated the presence of null alleles at locus LceCb in 12 sites. We therefore excluded this locus from all further analyses. Without locus LceCb, there was no strong evidence of deviations from HWE within populations. Although 29 of 423 single‐locus tests within populations were significant at *α *= 0.05, this is close to the approx. 21 significant tests expected by chance, and the deviations were spread erratically over loci and populations, including similar numbers of heterozygote excesses and deficits. No deviation was significant after Bonferroni correction. Accordingly, only four of 47 multilocus estimates of *F*_IS_ differed significantly from 0 at *α *= 0.05, and none of these deviations was significant at a Bonferroni‐corrected *α* of 0.001 (0.05/47 sites) (Table 1). There was no evidence of linkage disequilibrium among loci.

Sample sizes and genetic diversity statistics for all collection sites are summarized in Table 1. Expected heterozygosities ranged between 0.639 and 0.756, and the global *F*_ST_ estimate was 0.028 (0.022–0.036 95% C.I.). Table 1 also contains the estimates of *N*_e_ obtained with NeEstimator. These are generally very high with most values in the hundreds or thousands. For many samples, *N*_e_ was estimated as infinite and the upper confidence limit reached infinity in the majority of cases. The lowest estimates tended to come from the river Limmat, which was the only river for which estimates <100 were obtained (sites L22, L24 and L27).

### Bayesian clustering

The DIC plot indicated four as the most likely number of genetic clusters present in our chub samples. These clusters corresponded well to the populations in the four rivers we sampled from above their confluence (Fig. 1; for individual cluster membership coefficients, see Fig. S3). Samples from the river Rhine downstream of the confluence of the four rivers show admixture of the four clusters up until the most downstream site, whereas mixing upstream of confluences appears very restricted (Fig. 1).

### General effects of barriers

The IBD plot shows a monotonic positive relationship between pairwise *F*_ST_ and distance with increasing scatter (Fig. S4A). This corresponds to a case I relationship as defined by Hutchison and Templeton (1999) and is indicative of a regional equilibrium between gene flow and drift. Genetic differentiation increased with increasing waterway distance between sampling sites (Mantel *R *=* *0.595, *P* < 0.001) as well as with increasing numbers of barriers separating the sites (Mantel *R *=* *0.617, *P* < 0.001; Fig. S4B). Despite our efforts to break the collinearity when choosing sampling sites, waterway distances and barrier counts between sites were positively correlated with *R *=* *0.827. However, the partial Mantel tests indicated that the number of barriers between sites is a somewhat better predictor of genetic differentiation than distance *per se* (barrier count corrected for distance: *R *=* *0.278, *P *=* *0.002; distance corrected for barriers: *R *=* *0.190, *P *=* *0.048).

### Effects of different barrier types

The best‐supported model predicting pairwise *F*_ST_ between samples (model iii, AIC weight* *= 0.550, Table 2) included barriers in the predictors in addition to distance and distinguished between barriers with and without fishpasses, the latter including the Rhine falls. The second‐ranked model (model vi, AIC weight 0.290) additionally distinguished the Rhine falls from all other barriers without fishpass. The other models had a Δ AIC > 2 from the best‐supported model. Most notably, the model including distance only (model i, Δ AIC* *= 10.657, AIC weight 0.003) and the model not distinguishing between different types of barriers (model ii, Δ AIC* *= 8.857, AIC weight 0.007) received virtually no support from the data. These results provide strong evidence that barriers influence the genetic structure of chub in the large Swiss lowland rivers and that fishpasses appear to mitigate the negative effects of barriers on population connectivity. The parameter estimates from the best‐supported model iii allow gauging the effects of barriers with and without fishpass relative to free‐flowing stretches of river. According to this model, the effect of a barrier without fishpass is equivalent to approx. 102 km of uninterrupted river distance, while barriers with fishpass correspond to approx. 12 km. These values are in a similar range when calculated from the second‐ranked model vi (114 and 20 km, respectively), which assigns an even stronger effect to the only long‐standing natural barrier, the Rhine Falls, corresponding to approx. 220 km of uninterrupted river. Unsurprisingly, commonality analysis of the two best‐supported models showed substantial contributions of all predictors via common effects, reflecting the multicollinearity of distance and the numbers of different barriers between sites, but it also showed that in both models the barriers without fishpasses had the largest unique effect on genetic differentiation (Table 3a).

**Table 2:** Results of the model selection procedure based on AIC to assess the relative support of six candidate linear regression models predicting pairwise *F*_ST_ between chub samples from the Rhine drainage in Switzerland

Model		Slope	*R* ^2^	AIC	Δ AIC	AIC weight
(i)	Distance	2.731E‐4***	0.354	−216.549	10.657	0.003
(ii)	Distance	1.243E‐4***	0.404	−218.349	8.857	0.007
	All barriers	1.974E‐3***				
(iii)	Distance	1.122E‐4***	0.527	−227.206	0.000	0.550
	Barriers with fish bypass	1.392E‐3***				
	Barriers without fish bypass (including Rhine falls)	1.149E‐2***				
(iv)	Distance	2.319E‐4***	0.423	−219.848	7.358	0.014
	Rhine falls	1.878E‐2***				
(v)	Distance	3.904E‐5*	0.498	−224.423	2.783	0.137
	Rhine falls	2.476E‐2***				
	All barriers (excl. Rhine falls)	2.458E‐3***				
(vi)	Distance	8.191E‐5***	0.534	−225.929	1.277	0.290
	Rhine Falls	1.808E‐2***				
	Barriers with fish bypass	1.720E‐3***				
	Barriers without fish bypass	9.402E‐3***				

*Indicates *P *=* *0.051, ***indicates *P *<* *0.001.

**Table 3:** Commonality analysis of the best‐supported regression models (see Table 2) predicting genetic differentiation expressed as *F*_ST_ (a) and genetic diversity expressed as AR (b). Unique refers to each predictor's unique effect and Common refers to the sum of effects in common with other predictors in the model. Total is the sum of unique and common contributions to the explained variance in the response variable

Predictor	*R* ^2^	*B*	*P*	Unique	Common	Total	% of *R* ^2^
(a) Genetic differentiation (*F*_ST_)							
Model iii (AIC weight* *= 0.550)	0.527						
Distance		1.122E‐4	<0.001	0.019	0.334	0.354	67.1
Barriers with fish bypass		1.392E‐3	<0.001	0.024	0.268	0.292	55.4
Barriers without bypass (incl. Rhine Falls)		1.149E‐2	<0.001	0.162	0.149	0.311	59.0
Model vi (AIC weight* *= 0.290)	0.534						
Distance		8.191E‐5	<0.001	0.009	0.345	0.354	66.2
Rhine Falls		1.808E‐2	<0.001	0.048	0.146	0.194	36.3
Barriers with fish bypass		1.720E‐3	<0.001	0.031	0.261	0.292	54.7
Barriers without fish bypass		9.402E‐3	<0.001	0.062	0.176	0.239	44.7
(b) Genetic diversity (AR)							
Model vi (AIC weight* *= 0.853)	0.548						
Distance		0.005	0.072	0.037	0.060	0.096	17.6
Rhine Falls		0.349	0.227	0.016	0.020	0.037	6.7
Barriers with fish bypass		−0.109	0.001	0.127	0.146	0.273	49.8
Barriers without fish bypass		−0.544	<0.001	0.195	−0.031	0.165	30.0

**Figure 2:**
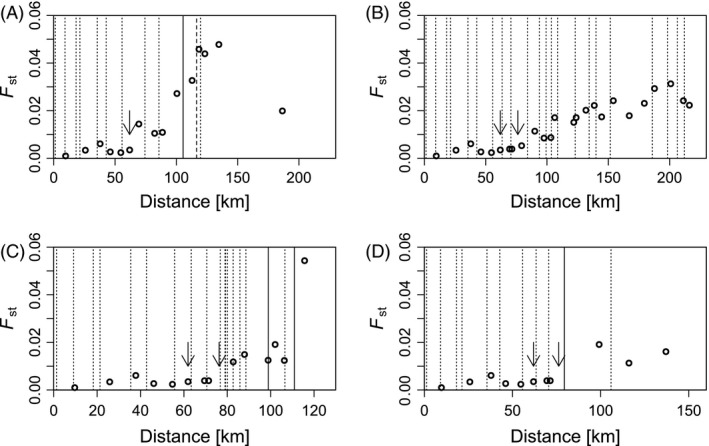
*F*_ST_ plotted against distance along the rivers Rhine (A), Aar (B), Limmat (C) and Reuss (D) from the most downstream sampling site in the Rhine, R29 (Fig. 1). Dotted lines are barriers with fish bypass, solid lines are barriers without fish bypass, and the dashed line represents the Rhine Falls. Confluences are indicated by arrows.

To visualize the effects of barriers in more detail, we have plotted the genetic differentiation (*F*_ST_) from the most downstream site (R29, Fig. 1) along each of the four rivers as a function of distance and added the position and type of barriers to these plots (Fig. 2). Although the effects are not strikingly obvious, more rapid increases of genetic differentiation tend to be associated with a high density of barriers (Fig. 2C) or with barriers that lack a fishpass (Fig. 2A,C,D).

**Figure 3:**
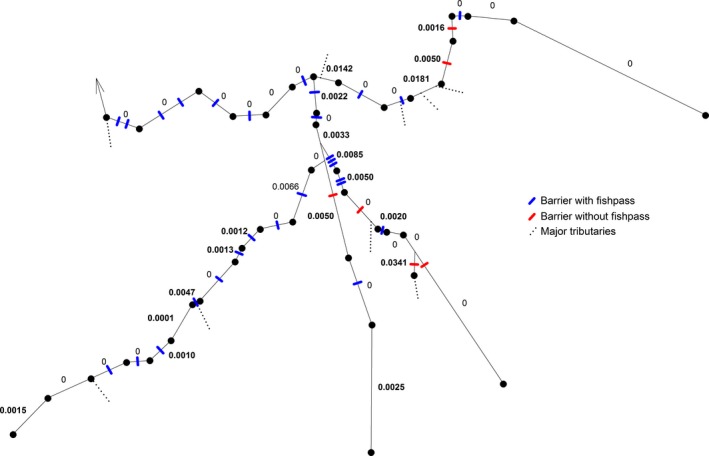
Genetic distance according StreamTree is mapped onto stream sections between the sampling sites. On the river network are sampling sites marked by black dotes, barriers with fishpass as blue bars, without fishpass as red bars, inflow major tributaries are symbolized by dashed lines, and the numbers are the genetic distance values.

With an *R* ^2^ of 0.836, the STREAMTREE model provided a fit to the actual genetic differentiation in the river network that, according to the authors of the algorithm (Kalinowski et al. 2008), is not very good and adverts to caution in interpreting the results. We report the results nevertheless because they provide an alternative visual representation of the resistance to migration in these rivers (Fig. 3) and because it highlights a potential additional factor contributing to genetic differentiation that was not considered in any other analyses. The STREAMTREE analysis predicted a genetic distance of 0 for more than half of all river sections considered, and although the nonzero values tended to be assigned to sections with a high density of barriers or fishpass‐free barriers, the match was not very good (Fig. 3). In particular, two of the highest values were assigned to sections without barriers but where major tributaries run into the Rhine river (from site R35 to R36 and R38 to R39, see Figs 1 and 3). The highest value was assigned to a section comprising a barrier without fishpass (short section below site L27, see Figs 1 and 3), yet this section is not part of the main course of the river Limmat. It represents the lowest part of an important tributary (river Sihl) from which sample L27 was taken upstream of the weirs separating the Sihl from the Limmat. Although certainly to be taken with caution, these observations suggest that immigration from unsampled tributaries may also contribute to genetic differentiation of chub along the main rivers of the upper Rhine catchment.

There is also correlative evidence that hydroelectric power plants and weirs affect the upstream decline of AR in chub populations. By far best‐supported in our set of candidate models (AIC weight* *= 0.853) was the most complex model vi that fitted separate effects for distance, the Rhine falls, barriers with fishpasses and barriers without fishpasses (Table 4). In this model, the number of both types of man‐made barriers between the most downstream point of the Rhine in Switzerland and the sampling sites have significantly negative effects on AR, but the effect of barriers without fishpass is about fivefold stronger (Table 4). Again, commonality analysis showed that barriers without fishpass make the largest unique contribution to the explained variance in AR, followed by barriers with fishpass (Table 3b). The estimated effect of the Rhine falls was nonsignificant but positive in this model, presumably just reflecting that the three samples from the Rhine above the Rhine falls had a higher allelic richness on average than most samples from the other rivers. More interestingly, the effect of distance in this model, which accounts for the effects of barriers, was also positive, although not significantly so.

**Table 4:** Results of the model selection procedure based on AIC to assess the relative support of six candidate linear regression models predicting allelic richness (AR) of chub in the Rhine drainage as a function of distance upstream from the most downstream point of the Rhine in Switzerland and the number of differently categorized barriers along this distance

Model		Slope	*R* ^2^	AIC	Δ AIC	AIC weight
(i)	Distance	−0.004*	0.096	88.758	26.543	0.000
(ii)	Distance	0.009**	0.440	68.277	6.062	0.041
	All barriers	−0.151***				
(iii)	Distance	0.009**	0.481	66.698	4.483	0.091
	Barriers with fish bypass	−0.152***				
	Barriers without fish bypass (including Rhine falls)	−0.322**				
(iv)	Distance	−0.004*	0.153	87.726	25.511	0.000
	Rhine falls	0.478				
(v)	Distance	0.009**	0.440	70.273	8.058	0.015
	Rhine falls	−0.134				
	All barriers (excl. Rhine falls)	−0.150***				
(vi)	Distance	0.005	0.548	62.215	0.000	0.853
	Rhine falls	0.349				
	Barriers with fish bypass	−0.109**				
	Barriers without fish bypass	−0.544***				

*Indicates *P *<* *0.05, **indicates *P *<* *0.01, ***indicates *P *<* *0.001.

**Figure 4:**
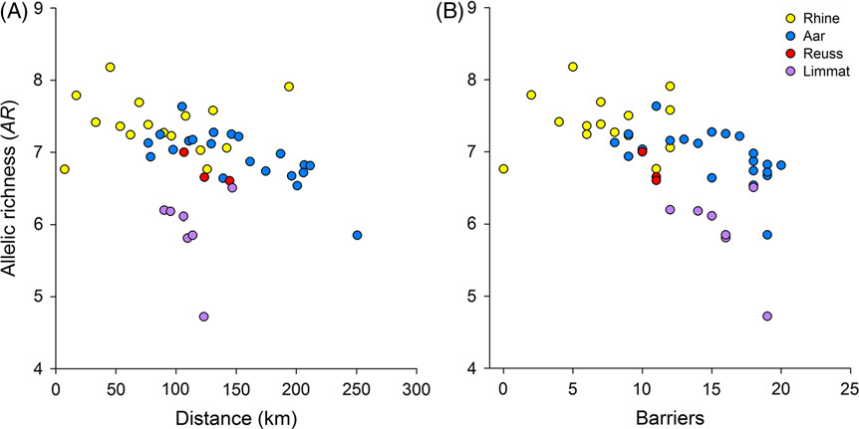
Plots of allelic richness against waterway distance (A) and the number of barriers (B) between the point where the Rhine river leaves Switzerland and the sampling sites. Symbol colours indicate rivers: yellow: Rhine; blue: Aar, red: Reuss, purple: Limmat.

Figure 4 illustrates the role of barriers. Allelic richness is better predicted by the number of barriers (*β *= −0.069, *R* ^2^ **= 0.307, *F*_1,45_ = 19.924, *P *<* *0.001) than by distance (*β *= −0.004, *R* ^2^ **= 0.096, *F*_1,45_ = 4.789, *P *=* *0.034), and this is to a large extent due to the samples from the river Limmat (Fig. 4, purple symbols). These are relatively close to the downstream sites of the Rhine river but separated by many barriers (Fig 1), including two without fishpasses (see Fig. 2C). Note that the river Limmat also produced the lowest estimates of *N*_e_.

## Discussion

### Genetic differentiation in a fragmented drainage

As expected for a mobile and abundant fish, the overall genetic differentiation of chub populations in the Swiss lowland rivers was low, with a global *F*_ST_ of only 0.028, and a large‐scale genetic structure shaped by drainage topology. The four genetic clusters identified by Bayesian clustering analysis corresponded well to the chub populations in the four rivers contributing to the drainage (Fig. 1). Similar observations were made in other fish species such as bullhead (Junker et al. 2012), bluehead sucker (Hopken et al. 2013) or brook trout (Kanno et al. 2011). We further observed significant effects of migration barriers on the chub's genetic structure. The Rhine Falls as the only long‐standing obstacle to migration seem to have a marked effect, but hydroelectric power stations and weirs also influence genetic differentiation detectably. Anthropogenic barriers thus contribute to the population genetic structure of the chub as also documented in other river‐dwelling fish species (Meldgaard et al. 2003; Hanfling and Weetman 2006; Leclerc et al. 2008; Raeymaekers et al. 2008; Blanchet et al. 2010; Dehais et al. 2010; Faulks et al. 2011; Horreo et al. 2011; Junker et al. 2012; Stelkens et al. 2012). Importantly, stations equipped with fishpasses impair population connectivity less than those without fishpasses. Depending on the model used (see Table 2), we estimated that isolation by barriers equipped with fishpasses on average corresponds to about 12 km (model iii) or 20 km (model vi) of uninterrupted waterway distance, markedly less than by barriers without fishpasses, estimated as 102 km and 114 km, respectively. For comparison, in a study of chub in the French river Durance, it was estimated that each dam adds a virtual distance of 34 km in terms of genetic differentiation (Dehais et al. 2010). An important additional insight from the present study is that fishpasses do indeed mitigate the negative effects of hydroelectric power stations on population connectivity, but that they do not annihilate this effect. The effect of barriers with fishpasses was significant in both of the best‐supported models predicting pairwise *F*_ST_ (Table 2). Notably, this concerns a fish that due to its size and swimming ability is predisposed to traverse fishpasses readily and that occurs in huge populations (Zaugg et al. 2003). The chub is indeed the only fish species that has been observed to pass all fishpasses present in our study area (Guthruf 2006, 2008). Thus, the negative effects on population connectivity of dams and hydroelectric power stations – even when equipped with fishpasses – are likely to be more pronounced for many other fish species having lower dispersal abilities. That the permeability of many fishpasses in Switzerland is insufficient was noted by Guthruf (2006), because not all size classes of fish and species were able to pass. A recent observation from the hydroelectric power station in Rheinfelden on the Rhine river (the first barrier upstream of site R30 in Fig. 1) supports the notion that most fishpasses are not as effective as they could be. In 2010, that is during our sampling campaign, this power station was equipped with a new fishpass of an improved design (a more or less naturally structured fishpass stream with high discharge). Counts revealed an upstream migration of approx. 40 000 fish from 33 species in one season, including species known as poor swimmers such as bullheads (*Cottus gobio*) (Energiedienst/PFA 2013). This is approximately four times more individuals than in the best‐frequented of the other fishpasses in our study area (Guthruf 2006, 2008), illustrating that there is indeed much room for improvement of the existing structures aiding fish migration across artificial barriers.

Specific recommendations for particular barriers are more difficult to make because the shallow population structure overall required a global analysis to detect their influence on population structure. Nevertheless, inspection of Fig. 2 suggests that some barriers such as the fishpass‐free power station at the bottom of the river Reuss (Fig. 2D) or the most upstream weir without fishpass in the river Limmat (Fig. 2C) are particularly influential. Their equipment with a fishpass would thus likely have a substantial positive effect on longitudinal connectivity of fish populations, and possibly also on effective population size, which tended to be lower in the river Limmat compared to other rivers. However, it is important to consider whether such measures would not conflict with other conservation goals. Nowadays, we are in the unfortunate situation that man‐made barriers can be the only thing protecting upstream reaches of a river from the invasion of nonindigenous species such as the Black Sea gobies or the North American crayfish species currently expanding along the Rhine river (Leuven et al. 2009; Mombaerts et al. 2014), or to shelter autochthonous from stocked fish populations (Fausch et al. 2009). In this context, re‐establishing the longitudinal permeability of river networks for aquatic organism to a state prior to human influence may not be desirable.

The STREAMTREE model suggested that unsampled tributaries may also contribute to the genetic differentiation of chub along the main rivers we sampled. Considering that Bayesian clustering distinguished the populations from the four largest rivers of the drainage rather well (Fig. 1), it is reasonable to assume that populations from smaller rivers of the same drainage would also exhibit some genetic differentiation. Their influence is likely to act in combination with barriers to migration. If fish from such tributaries disperse into the main river but contribute predominantly to populations downstream of the confluence because obstacles prevent upstream movement, it is easy to see how river sections receiving major tributaries affect differentiation along the main stream and are assigned larger genetic distances by the STREAMTREE algorithm.

### Genetic diversity

An upstream decline in genetic diversity is generally expected in organisms inhabiting a dendritic river network because of the accumulation of allelic diversity below confluences of tributaries containing genetically differentiated populations (Morrissey and de Kerckhove 2009; Paz‐Vinas and Blanchet 2015). That is indeed what we observed when we quantified genetic diversity as allelic richness. However, our analyses indicated that the upstream decline of allelic richness is exacerbated by man‐made barriers. This was particularly obvious from the comparatively low allelic diversity of chub from the sampling sites along the river Limmat, which are relatively close to the most downstream sites we considered but separated by many barriers (Fig. 2C). Note that based on the (uncertain) estimates, we obtained with NEESTIMATOR, *N*_e_ of chub in the Limmat might also be lower than in the other rivers (Table 1).

In the best‐supported model predicting AR, which accounted for the negative effect of man‐made barriers, the parameter estimate for distance was in fact slightly (and nonsignificantly) positive (Table 4). A possible explanation for this observation is that the midland rivers we studied all pass through large lakes upstream of our sampling sites. We suspect that the large lake populations of chubs may act as a reservoir of allelic diversity that feeds into the lowland rivers from above and partially compensates for the upstream loss that is otherwise observed along these fragmented rivers. This conjecture is tentatively supported by the two population samples we obtained from Lake Constance (R44 in Fig. 1) and Lake Zurich (L28 in Fig. 1). They are represented in Fig. 4A by the most upstream yellow and purple points, respectively, which show a high allelic diversity compared to the general trends along the rivers Rhine and Limmat.

## Conclusions

We show that the chub in the Swiss lowland rivers has a shallow population structure that is shaped by drainage topology, but also significantly affected by man‐made barriers to migration, most of which are hydroelectric power stations. From a management perspective, it is important to note that the fishpasses installed at many of these stations do indeed improve fish population connectivity across the barrier. Nevertheless, even barriers with fishpasses have a detectable effect on genetic differentiation, in a fish that is relatively large and mobile and copes equally well with lotic and lentic environments, that is a species that should be among those least susceptible to habitat fragmentation. The negative effects of river fragmentation are likely to be more severe for many other river‐dwelling fish species. Fortunately, improvements in the design of fishpasses show great promise in reducing these effects further, as seen for the Rheinfelden power station mentioned above. It is to be hoped that similar improvements will soon be realized for other barriers as well.

## Data archiving statement

Data available at Dryad Digital Repository: http://dx.doi.org/10.5061/dryad.n41nk.

## Supporting information


**Figure S1.** Map showing the location of all barriers included in Supplementary Table S1.

**Figure S2.** Neighbourhood Diagram used in the Bayesian clustering analysis (TESS 2.3.1 output).

**Figure S3.** Genetic cluster affiliations of individual fish estimated at *K*_max_ = 4 in TESS 2.3.1 and visualized with DiSTRUCT.

**Figure S4.** Isolation‐by‐distance and ‘isolation‐by‐barriers’ of chub in the Swiss Lowland rivers depicted as linear regression plots of pairwise *F*_ST_ against waterway distance (A) and the number of barriers (B) between sampling sites.

**Table S1.** Table reporting ages of the barriers and their fishpasses.
