# Supplementary material for: Fish population genetic structure shaped by hydroelectric power plants in the upper Rhine catchment
Source: Evol Appl. 2016 Jan 8;9(2):394–408. doi: 10.1111/eva.12339 (PMC4721079; doi:10.1111/eva.12339)
Supplement: Supplementary file 1 — Figure S1. Map showing the location of all barriers included in Supplementary Table S1. [file EVA-9-394-s001.pdf]

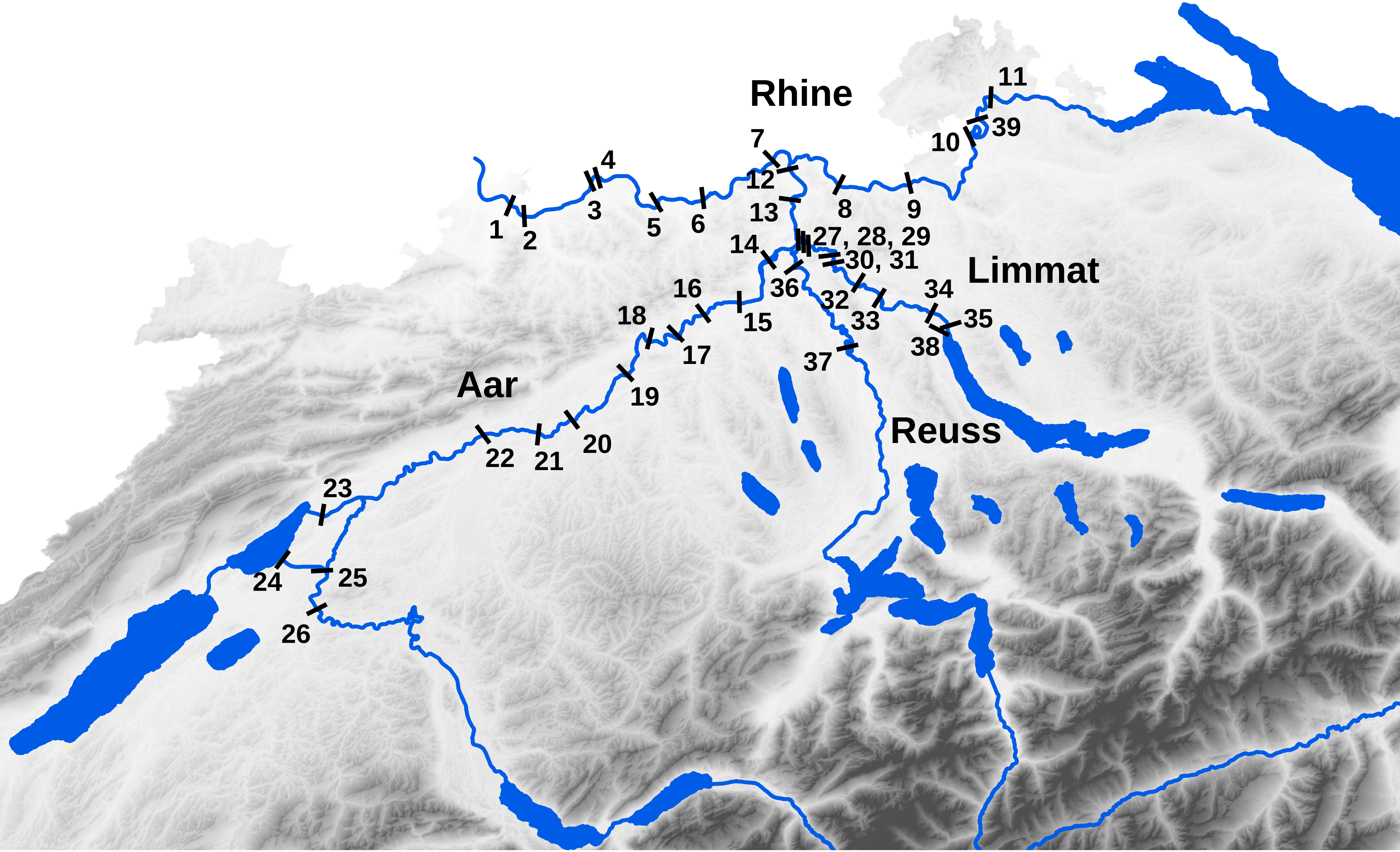

Fig. S1 Black bars represent barriers and the numbers correspond to the barrier numbers in Table S1.
