# Supplementary material for: Fish population genetic structure shaped by hydroelectric power plants in the upper Rhine catchment
Source: Evol Appl. 2016 Jan 8;9(2):394–408. doi: 10.1111/eva.12339 (PMC4721079; doi:10.1111/eva.12339)
Supplement: Supplementary file 2 — Figure S2. Neighbourhood Diagram used in the Bayesian clustering analysis (TESS 2.3.1 output). [file EVA-9-394-s002.pdf]

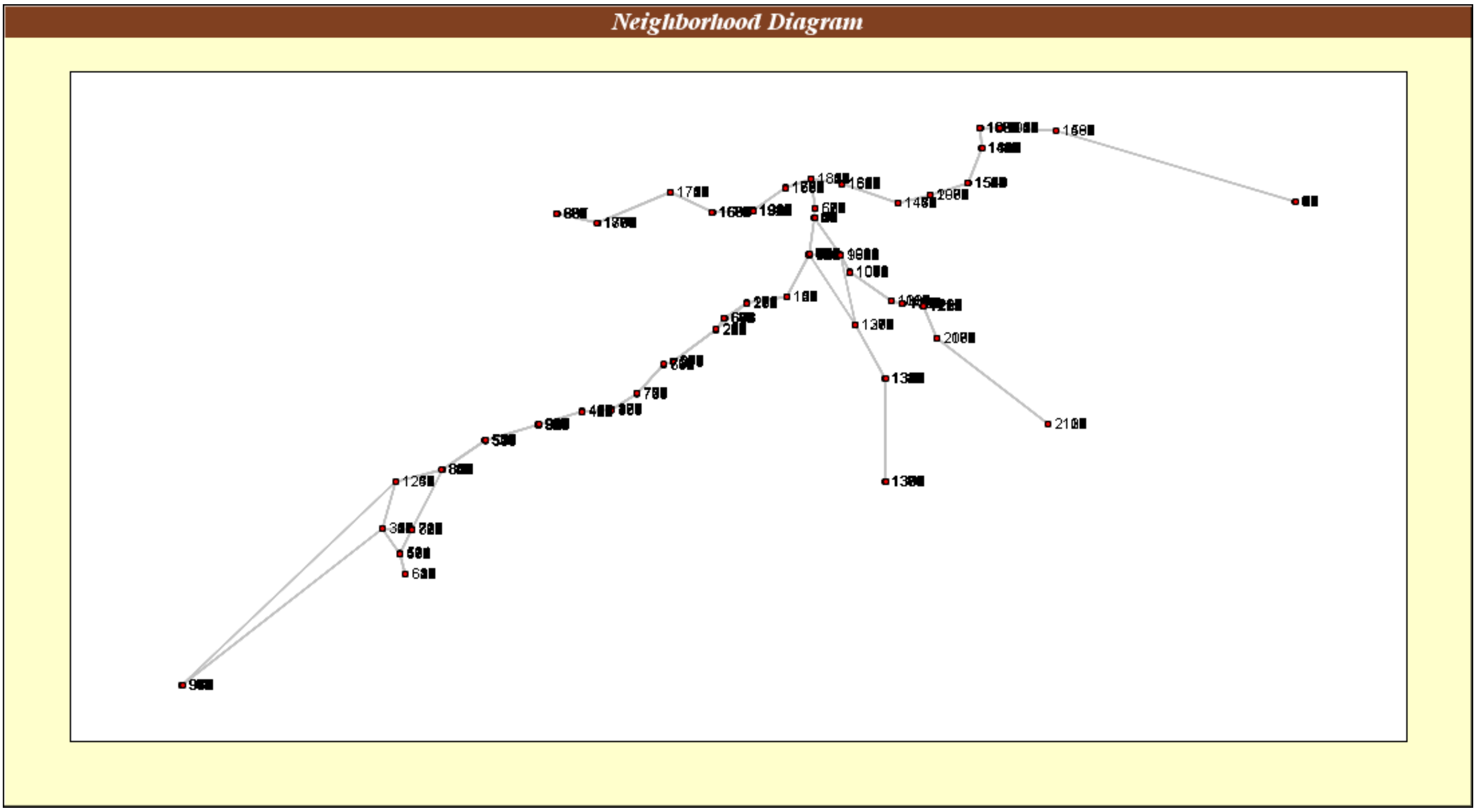

Fig. S2 Tess 2.3.1 neighborhood Diagram output, showing the network construction
