# Supplementary material for: Fish population genetic structure shaped by hydroelectric power plants in the upper Rhine catchment
Source: Evol Appl. 2016 Jan 8;9(2):394–408. doi: 10.1111/eva.12339 (PMC4721079; doi:10.1111/eva.12339)
Supplement: Supplementary file 3 — Figure S3. Genetic cluster affiliations of individual fish estimated at K max = 4 in TESS 2.3.1 and visualized with DiSTRUCT. [file EVA-9-394-s003.pdf]

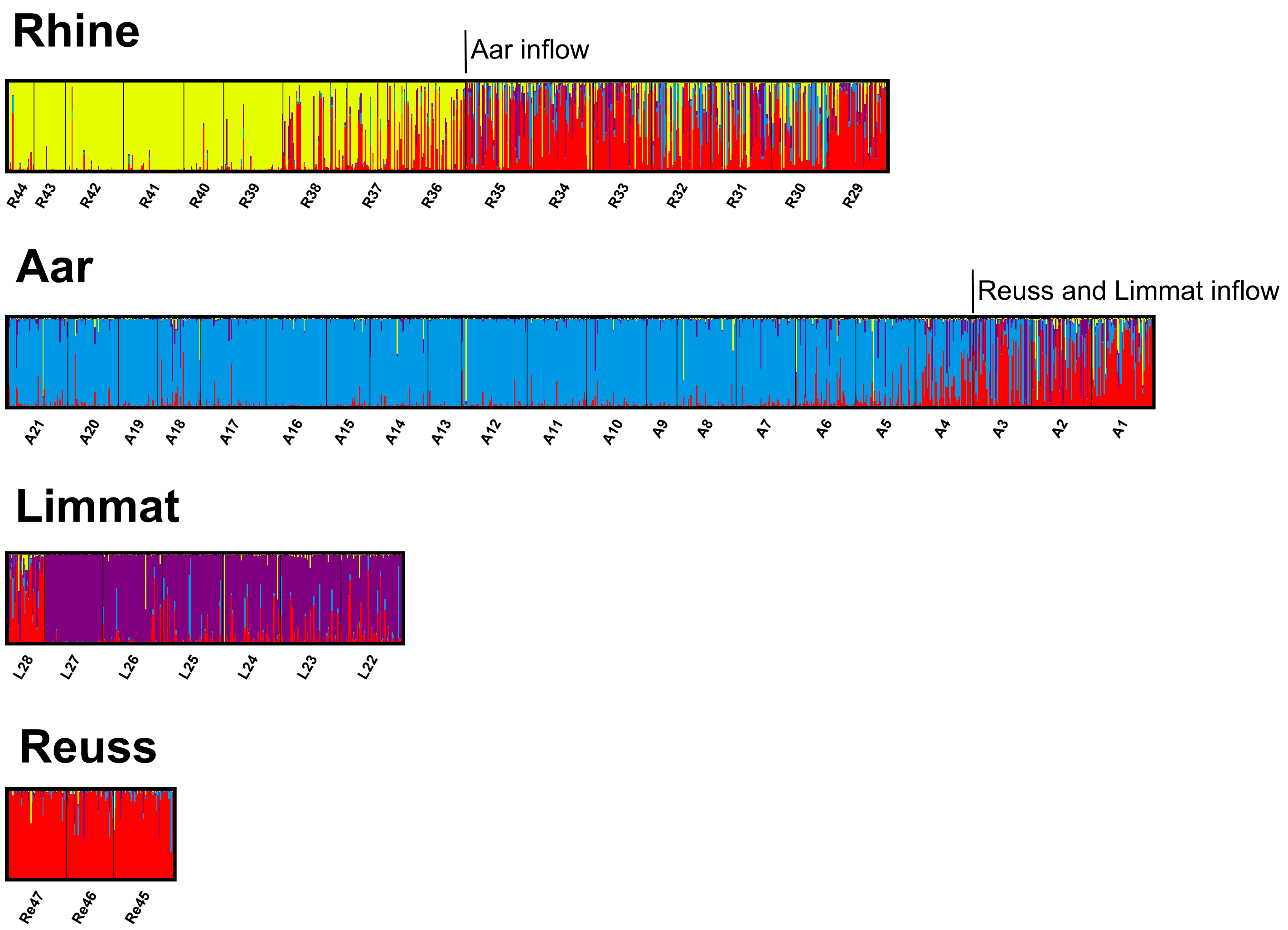

Fig. S3 TESS 2.3.1 individual cluster affiliation visualized with DiSTRUCT.
For the geographic distribution of the sites see Fig.1 and coordinates Table 1.
