# Supplementary figures and images for: Fish population genetic structure shaped by hydroelectric power plants in the upper Rhine catchment
Source: Evol Appl. 2016 Jan 8;9(2):394–408. doi: 10.1111/eva.12339 (PMC4721079; doi:10.1111/eva.12339)

(A)

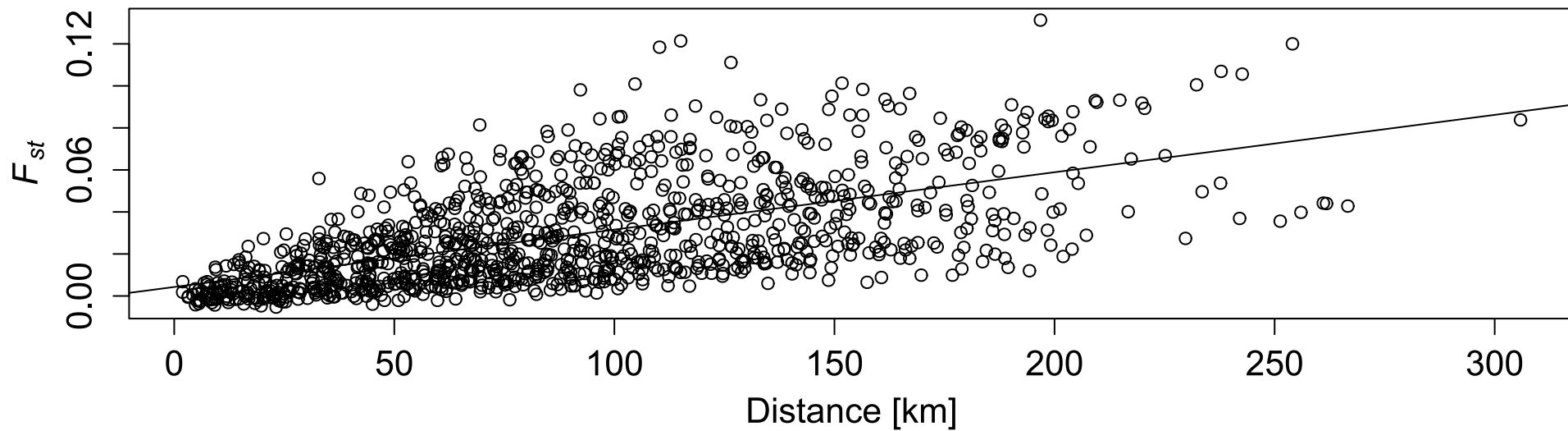

(B)

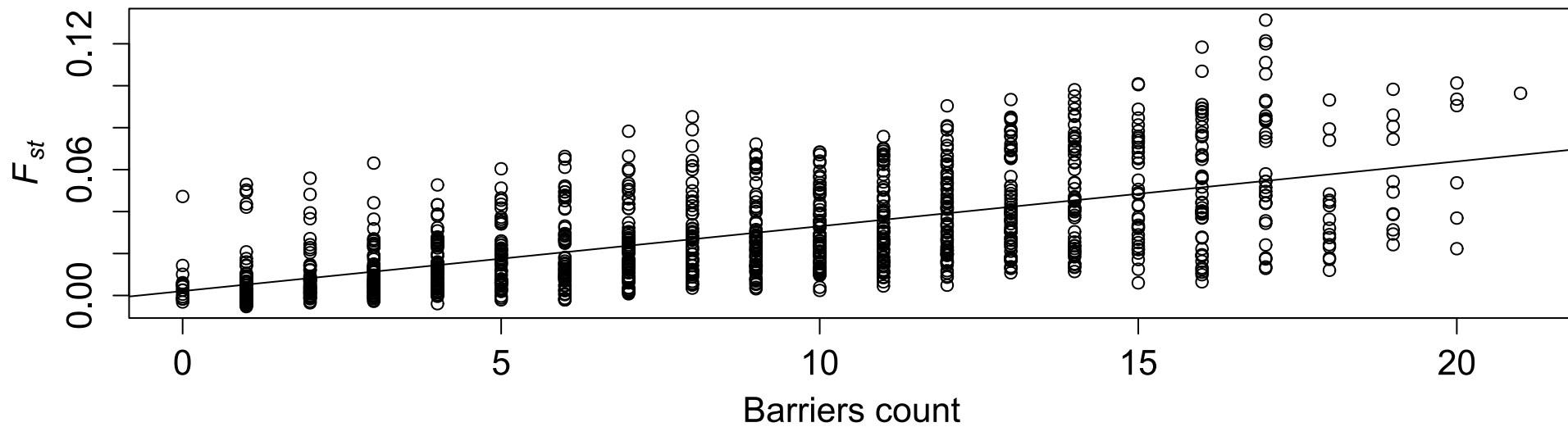

Supplement: Supplementary file 4 — Figure S4. Isolation‐by‐distance and ‘isolation‐by‐barriers’ of chub in the Swiss Lowland rivers depicted as linear regression plots of pairwise F ST against waterway distance (A) and the number of barriers (B) between sampling sites. [file EVA-9-394-s004.pdf]
