# Supplementary material for: Fish population genetic structure shaped by hydroelectric power plants in the upper Rhine catchment
Source: Evol Appl. 2016 Jan 8;9(2):394–408. doi: 10.1111/eva.12339 (PMC4721079; doi:10.1111/eva.12339)
Supplement: Supplementary file 5 — Table S1. Table reporting ages of the barriers and their fishpasses. [file EVA-9-394-s005.pdf]

Table S1 Ages of all barriers and their fish bypasses (if present) considered in this study. Numbers of barriers correspond to Figure S1.

Barrier number	Launch of electricity production	Fish bypass	Bypass construction date
Hydroelectric power stations			
1	1955	yes	No publicly availabe information
2	1912	yes	1912
3	1898	yes	1898
4	1931	yes	< 1959
5	1966	yes	1966
6	1914	yes	1914
7	1933	yes	1933
8	1941	yes	1941
9	1920	yes	1920
10	1956	no	Bypass planned within next 10 years
11	1964	yes	1964
12	1935	yes	1952
13	1902	yes	1902
14	1953	yes	1953
15	1945	yes	1949
16	1929	yes	1929
17	1893	yes	1893
18	1917	yes	1917
19	1896	yes	1896
20	1896	yes	1896
21	1970	yes	No publicly available information
22	1970	yes	1982
23	1939	yes	No publicly available information
24	1900	yes	No publicly available information
25	1968	yes	1995
26	1963	yes	No publicly available information
27	1861	yes	No publicly available information
28	1902	yes	No publicly available information
29	1896	yes	No publicly available information
30	1892	yes	2006
31	1909	yes	1909
32	1933	no	Bypass opened in 2010 (after sampling)
33	1933	yes	1933
34	1898	yes	No publicly available information
35	1877	no	Bypass opened in 2010 (after sampling)
36	1830	no	Bypass planned within next 10 years
37	1975	yes	1975
Weir			
38	1927	no	Replacement by block ramp planned
Rhine Falls			
39	-	no	Formed 14'000 to 17'000 years ago
